# Improving Uptake and Adherence to 17-Hydroxyprogesterone Caproate in Non-Hispanic Black Women: A Mixed Methods Study of Potential Interventions from the Patient Perspective

**DOI:** 10.1089/biores.2019.0010

**Published:** 2019-10-23

**Authors:** Sarahn M. Wheeler, Kelley E.C. Massengale, Katelyn P. Blanchard, Thelma A. Fitzgerald, Teresa Swezey, Geeta K. Swamy, Amy Corneli

**Affiliations:** ^1^ Division of Maternal and Fetal Medicine, Department of Obstetrics and Gynecology, Duke University School of Medicine, Durham, North Carolina.; ^2^ Diaper Bank of North Carolina, Durham, North Carolina.; ^3^ Duke Clinical Research Institute, Durham, North Carolina.; ^4^ Department of Population Health Sciences, Duke University School of Medicine, Durham, North Carolina.

**Keywords:** 17-hydroxyprogesterone caproate, 17-OHPC, 17-P, adherence, disparity, intervention, premature birth, preterm birth, progesterone

## Abstract

Women with a history of a preterm birth (PTB) are at high risk for recurrence. Weekly 17-hydroxyprogestrone caproate (17-P) injections can reduce the risk of recurrence in women with prior spontaneous PTB. PTB occurs disproportionately in non-Hispanic black (NHB) women, and uptake and adherence to 17-P among NHB women are lower compared to women in other racial/ethnic groups. Evidence-based interventions to improve 17-P uptake and adherence that incorporate women's perceptions and preferences are needed. Our objective was to identify women's perspectives and preferences for interventions to promote uptake of and adherence to 17-P, particularly among NHB women. We conducted an exploratory sequential mixed methods study using focus group discussions (FGDs), a survey, and in-depth interviews (IDIs). We recruited women with a history of PTB who self-identified as NHB for the FGDs and IDIs. Survey participation was open to any woman with a history of PTB regardless of their race and ethnicity. Women could only participate in one of the three data collection activities. Transcripts from the qualitative focus groups and in-depth interviews were analyzed using applied thematic analysis. Descriptive statistics was used to analyze the quantitative survey. Eighty-two women participated in the study (FGDs [*n* = 7], surveys [*n* = 60], and IDIs [*n* = 15]). Suggested interventions were separated into two categories: (1) clinic-based interventions (i.e., interventions delivered during the clinical encounter) and (2) community-based interventions (i.e., interventions delivered outside of the clinical encounter). Clinic level interventions included improved clinic access and scheduling, same-day appointments, appointment reminders, making the clinic experience more comfortable for patients, and encouragement from providers. Interventions at the community level included increased 17-P awareness among support persons, employers, and community members and administration of 17-P outside the clinic setting. Our findings offer multiple potential interventions that could improve uptake of and adherence to 17-P for PTB prevention among NHB women. These proposed interventions have the potential to mitigate barriers to 17-P and narrow the disparity in PTB rates. Given the alarming and increasing rates of prematurity and PTB disparities, it is imperative to test, refine, and incorporate effective interventions into clinical practice. Our findings provide insights from patients that can help shape such interventions.

## Background

Preterm birth (PTB), delivery before 37 weeks gestation, is the leading cause of neonatal morbidity and mortality.^1^ Dramatic racial disparities persist with a 49% higher incidence of PTB in non-Hispanic black (NHB) compared to white women.^2^ The risk of PTB is highest among women with a history of PTB.^3,4^ Weekly 17-hydroxyprogestrone caproate (17-P; AMAG Pharmaceutical, Waltham, Massachusetts) injections can reduce the risk of recurrent PTB in women with a history of spontaneous PTB.^3,5,6^

Despite the documented benefit of 17-P, in a recent study NHB women initiated 17-P later and missed doses more often than women in other racial and ethnic groups.^7^ Accepting the 17-P treatment recommendations (uptake) and adhering to the weekly treatment regimen (adherence) are necessary steps for achieving the benefit of 17-P.^8^ Evidence-based interventions to improve 17-P uptake and adherence are needed,^8^ especially those that incorporate women's perspectives and preferences. While research has identified numerous barriers to 17-P uptake and adherence at the provider and systems levels^9^ in which interventions can address, research describing NHB women's perspectives and preferences on 17-P interventions is missing. We conducted a mixed methods study among women at risk for PTB to identify potential interventions to promote uptake and adherence to 17-P.

## Methods

### Study design

We used an exploratory sequential mixed methods study design.^10^ The data collection activities were (1) focus group discussions (FGDs), (2) a survey, and (3) in-depth interviews (IDIs). The study was approved by the Duke Health Institutional Review Board (Pro00084334) on July 3, 2017, and all participants provided written or electronic informed consent before participation. Participants were recruited from August 2017 to April 2018 from a single, academic high-risk obstetrical clinic in Durham, NC.

#### Eligibility criteria and recruitment

Women were eligible to participate if they (1) had a current or recent (within the past 2 years) pregnancy during which they were clinically deemed eligible for 17-P (i.e., current singleton gestation, history of prior singleton spontaneous preterm delivery), (2) were recommended to initiate 17-P, and (3) were 20 weeks gestation or beyond. Women were eligible for participation irrespective of whether they accepted or rejected recommendations for 17-P. We excluded women deemed ineligible for 17-P or if they were not English literate.

Women of all races and ethnicities were eligible to participate in the survey. Our clinic provides care to any woman needing services, and therefore, an intervention delivered through our clinic would be available to all women irrespective of race and ethnicity. For the FGDs and IDIs, however, we exclusively recruited women who self-identified as NHB. We chose to focus the qualitative data collection on NHB women given the race disparities in PTB and 17-P utilization. Our ultimate goal was to use the qualitative findings to develop an intervention on improving 17-P uptake and adherence that was tailored for the specific preferences of NHB women but also likely acceptable to non-NHB women. Women were limited to participation in one of the data collection activities.

Women meeting the eligibility criteria were approached during their routine clinic visit. Women meeting criteria for the FGDs were scheduled for participation at one of three times offered in the morning, afternoon, or evening. Children were welcome at the FGDs. The survey was administered during the clinic visit using a tablet on the date of consent. IDI participants were offered to schedule an interview in person or on the phone. Of note, we also were able to offer “on-demand” interviewing on the date of consent for participants who elected for immediate participation.

#### Routine clinic procedures

The American Congress of Obstetricians and Gynecologists and Society for Maternal Fetal Medicine guidelines recommend that women with a history of spontaneous PTB receive intramuscular injections of 17-P starting between 16 and 20 weeks gestation continuing weekly until 36 weeks.^3,6^ At the time of data collection, 17-P eligible women at our clinic were identified and offered 17-P at their first prenatal appointment. Based on current North Carolina state insurance policies, women with public or no insurance must receive 17-P injections in the clinic because the medication is distributed to the clinic rather than individual patients. In the setting of private insurance, the medication can be dispensed to the patient, allowing the option for a family member to administer 17-P injections at home. After a clinic appointment is scheduled, women can view their appointments through Duke MyChart, an online and mobile patient portal in Epic's electronic health record. Appointment reminders are also sent through Duke MyChart, telephone, or postal mail.

#### Data collection

##### Demographic information

Baseline demographic and pregnancy data, including gestational age at participation (if currently pregnant) and receipt of 17-P, were collected from the electronic medical record.

##### Focus group discussions

We first conducted the FGDs. We chose to use FGDs to capitalize on the group dynamic to generate potential interventions to improve 17-P uptake and adherence. Participants described their suggestions for interventions that they believed would encourage more NHB women to initiate and adhere to the 17-P regimen. The FGDs were audio recorded, facilitated by a trained moderator, attended by a notetaker, and transcribed.

##### Survey

We developed a brief survey focused on gauging perceived acceptability and preferences of 17-P uptake and adherence interventions. Using a list of potential interventions identified from the FGDs, participants ranked their preferred top three interventions. The survey was administered using an electronic tablet during a clinic visit. The survey was conducted among women of all racial and ethnic groups.

##### IDIs

After a limited number of women participated in our three FGDs given group scheduling conflicts, we altered our approach to include IDIs so we could obtain more qualitative data from NHB women. We asked very similar questions in the IDIs as the FGDs, although we modified the question guide to focus on individual experiences and preferences rather than community perspectives. The IDIs were conducted by a trained interviewer in a private room within the clinic, audio recorded and transcribed.

### Analysis

Demographic characteristics of the participants are presented using descriptive statistics. Receipt of 17-P was measured as a binary outcome (yes/no), and receiving of a single dose was categorized as positive receipt.

For the qualitative data, we used applied thematic analysis to analyze the data.^11^ All FGDs and IDIs were transcribed verbatim. After reviewing all the transcripts, we developed a codebook of structural and emergent codes. Structural codes were first applied to the transcripts to organize participant responses based on the FGD/IDI guide questions. We then revised and expanded the codebook to include additional emergent codes after reading the IDI transcripts and applied them to the IDIs and to the FGDs when relevant.^12^ Emergent codes identified common themes among participant responses. Each FGD and IDI transcript was coded independently by two analysts. Inter-rater reliability procedures were conducted, and coding differences were resolved by discussing each instance until a mutual consensus was reached.^13^ While we were able to use a similar codebook to apply codes to the FGDs and IDI transcripts, we analyzed the FGD transcripts independently from the IDI transcripts and did not combine data. For both the FGDs and IDIs, we identified patterns among responses within individual codes and then looked for patterns across codes to identify salient themes. Finally, potential interventions identified within these themes were categorized by the environment in which the intervention would be implemented (clinic or community) and then summarized.

## Results

A total of 82 women participated: 7 participated in the FGDs (across three groups, *n* = 3, 2, and 2, respectively), 60 completed the survey, and 15 participated in the IDIs. Participant characteristics by data collection activity are presented in the Table 2. Suggested interventions were separated into two overarching categories: (1) clinic-based interventions and (2) community-based interventions (Table 1). We then present the most commonly suggested interventions in the FGDs and IDIs along with rankings based on the survey data. Illustrative quotes are presented below and in Table 2.

**Table 1.** Participant Characteristics by Data Collection Arm

	FGD (*n* = 7)	IDI (*n* = 15)	Survey (*n* = 60)
Self-identified race/ethnicity, *n* (%)			
Non-Hispanic black	7 (100)	15 (100)	27 (46)
Non-Hispanic white			20 (34)
Hispanic			8 (13)
Asian			2 (3)
Multiracial			5 (4)
Age, mean (SD)	32.6 (5.7)	31 (5.9)	29 (5.5)
Marital status, *n* (%)			
Single	6 (86)	10 (67)	31 (53)
Married	1 (14)	5 (33)	26 (44)
Divorced	0 (0)	0 (0)	2 (3)
Insurance status, *n* (%)			
Private	0 (0)	7 (47)	19 (34)
Medicaid/Medicare	7 (100)	8 (53)	36 (64)
Uninsured	0 (0)	0 (0)	1 (2)
Number of prior PTB, median (IQR)	1 (1–1)	1 (1–1.5)	1 (1–2)
Gestational age @ participation (in weeks)	22.71^a^	28.27^b^	25.52^c^
Receipt of 17-P	5/5	9 (60)	50 (85)

^a^ *n* = 5; excludes two women who participated postpartum.

^b^ *n* = 13; excludes two women who participated postpartum.

^c^ *n* = 56; excludes one women who participated postpartum and three missing data.

FGD, focus group discussion; IDI, in-depth interview; PTB, preterm birth.

**Table 2.** Summary of Patient-Proposed Interventions and Representative Quotes

Patient-proposed interventions	Representative quotes
Clinic-based interventions	
Improved clinic access and scheduling	“*…like if y'all open Monday through Friday from 8 o'clock to 5 o'clock and I'm getting off work at 6 o'clock then there is no way and I'm not going to miss my job for a shot.*”
Offer appointment reminders	“*I juggle so many things in a day and it be hard for me to remember.*”
Make the clinic experience more comfortable	“*…because when you come in like you see everybody else and sometimes they have significant others and if you're by yourself it's discouraging. Because you look at it like maybe I got to go through this by myself.*”
Provide encouragement	“*Maybe here and there like, one, two, three, about three visits down the line say,* ‘*hey, you know, good job with the 17-P, glad you're still getting them.*’”
Community-level interventions	
Increased 17-P awareness among support persons and employers	“*…so then I had to kind of like talk to my boss like,* ‘*look I have to take these shots every Thursday; can I get Thursdays off?*’ *And it took a while to get my schedule like that.*”
Increased community 17-P awareness	“*Yes, getting out into the community. Like you said, information; that's good. Not only just talking to the people who is at risk for having premature; talk to the people who is having full-term so that way they can you know, know and spread the word and stuff.*”
Administer injections outside of the clinic	“*Far as seeing if people that's you know basically just people just far away, if they could go to like their local health department or do the shots at home or if a nurse could come from the health department or you know.*”

### Clinic-based interventions

#### Improved clinic access and scheduling

The most commonly suggested clinic-based intervention in the FGDs and IDIs was extended clinic hours. FGD and IDI participants alike reported that clinic hours before 8 am or in the evening would allow them to receive injections without missing work. In addition to offering extended weekday hours, an IDI participant suggested limited weekend hours*:*

…maybe open a clinic on a Saturday just for shots. So in case people who work Monday thru Friday they can go Saturday for their shots or like maybe from like 10–12 or something like that for shots only for those who can't do it on a weekday.

In the survey, 14% of respondents ranked convenient clinic hours among their top three interventions that would facilitate uptake and adherence to 17-P.

Some IDI participants described that having appointments on the same day each week helped them remember appointments. An IDI participant explained, *“…I stay consistent. I come every Thursday so that way it won't be no mishaps.”* Another IDI participant suggested to have the 17-P appointments immediately following her provider appointment rather than having separate provider and injection appointments on the same day yet hours apart. More than half of survey respondents ranked “easy scheduling” among their top three most helpful interventions to maintain 17-P adherence.

#### Offer appointment reminders

Participants described the importance of appointment reminders within the context of their lives. One FGD participant said Duke MyChart, *“actually reminds me.”* An IDI participant said she avoids thinking about her 17-P appointments because the injections are painful, and therefore, the reminders were especially useful for her. Among survey respondents, 30% ranked appointment reminders through text message and 18% ranked telephone reminders among the top three interventions most likely to improve 17-P adherence.

#### Make the clinic experience more comfortable for patients

Both FGD and IDI participants described that feeling uncomfortable in the clinic environment influenced their adherence to 17-P. An IDI participant explained that seeing other patients in the waiting room with their partners or other support persons can be tough for someone without a support network. For another IDI participant, seeing mothers with their babies in the waiting room served as an emotional reminder of her own pregnancy loss. One IDI participant said that visiting any type of clinic environment was stressful, *“I get anxiety. Like if I'm coming to the doctor I'm like what if they tell me anything bad, what if they tell me bad news?”*

Another aspect of the overall clinic environment participants described was the variable amount of waiting time before receiving their injections. One of the FGD participants commented, “*it was times when I sit here and I was like frustrated.”* Among survey participants, 27% felt that a “short clinic wait time” to receive injections was one of the top three factors that would help them adhere to the weekly injection schedule.

#### Provide encouragement

During the IDIs, some participants indicated that having their health care providers take the time to provide encouraging words would positively impact their adherence to the 17-P regimen. An IDI participant specified that positive encouragement would acknowledge the effort taken to receive the injections. Forty percent of survey respondents ranked “provider reminders” about the importance of 17-P among their top three preferred interventions.

### Community-level interventions

#### Increase 17-P awareness among support persons and employers

FGD and IDI participants alike felt that with increased awareness, 17-P would be less foreign when they explained it to their family members and would help employers understand the need to make time for weekly injections. FGD and IDI participants advocated for brochures and information about 17-P they could share with others so that people in their social support networks could be supportive of them getting the injection. As one IDI participant explained, *“Well my mom, she's still confused about it. She's like ‘what is that’ and she asks me. She stopped, I think she stopped like last week, stopped asking me, ‘what you keep going there and getting a shot for?’”*

Other FGD and IDI participants described challenges scheduling 17-P injections around their work schedules. An IDI participant spoke with her supervisor about her need to schedule shifts around her 17-P injections, but lamented the conversation was challenging because her time off would affect her coworkers as well.

#### Increase community awareness of 17-P

FGD and IDI participants suggested that airing commercials on television and radio, advertising with banners on social media websites, and placing ads in magazines would increase awareness of 17-P among the general population. They also suggested sharing information at community events such as health fairs and local sporting events. An IDI participant described that because people may be afraid of visiting the doctor, reaching them in the community would be more effective. *“You know a lot of people don't like coming to the doctor so I think probably coming out into a community thing you know.”*

#### Administer injections outside of the clinic

Both IDI and FGD participants spoke about having injections outside of the clinic. Participants who had health care providers as family members or coworkers were receptive to the idea of receiving injections outside of the clinic, as the injections would be delivered by a trained professional. Concerns were expressed, however, about having nonmedical professionals give the injection, as described by one FGD participant who declined the option, *“I didn't trust my husband to do it. I trusted him but he is not a nurse and he's not trained, you know, he's not trained.”* Those participants who could not identify a friend or family member to administer the injection were interested in having a health care provider visit them at home instead. An IDI participant explained that sending health care providers to peoples' homes to administer injections would address barriers to visiting the clinic weekly. In addition, participants that lived far from the clinic were also interested in injections at another place in their own communities. Among survey respondents, 37% ranked “receiving injections at home or work” among the top three interventions most likely to encourage uptake and adherence.

## Discussion

Our findings offer multiple potential interventions that could potentially improve uptake of and adherence to 17-P for PTB prevention among NHB women. The suggested interventions focus on actions at the clinic and community levels. Clinic-level interventions included extended clinic hours, same-day appointments, appointment reminders, improvements to the clinic environment, and encouragement from providers. Interventions at the community level included increased 17-P awareness among support persons, employers, and community members and injections outside the clinic setting. These proposed interventions could potentially help address the patient, provider, and system level barriers identified in the comprehensive review on operationalizing six steps to 17-P utilization by Stringer et al.^8,9^

The most commonly suggested clinic-based intervention from the qualitative findings was extended clinic hours to include early morning, late evening, and weekend hours. There is evidence within vaccine literature suggesting that extended clinic hours could be an efficacious strategy.^14^ Goad et al. found that after-hour vaccine administration was commonly utilized and more popular among younger patients.^14^ The pregnant population also falls within a younger demographic further suggesting that extended clinic hours may be an efficacious intervention.

Perhaps the most challenging clinic-level interventions to enact are the suggested interventions around making the clinic environment more comfortable. For women lacking social support or with a tragic history of loss, attending clinic visits and seeing other patients with supportive partners or healthy babies may evoke anxiety. Eliminating this anxiety is challenging; however, our findings point to a need for modifications in our clinic space to provide more privacy within the waiting room. Universal social work consultation in 17-P eligible patients with nonadherence triggering follow-up consultation may also be impactful.

For community-based interventions, the qualitative findings suggest that providing injections outside of the clinic environment may be particularly effective at improving uptake and adherence. A subcutaneous auto-injection formulation for 17-P was recently FDA approved that may be administered with limited training. This dosing formulation may provide an additional avenue to facilitate injections outside the clinic setting.

Although we present many important insights about potential interventions to improve uptake and adherence to 17-P, our findings must be viewed in light of some important limitations. First, the sample size of the focus group is very low. We were able to overcome this limitation, however, by augmenting our FGD findings with data with IDIs. Although the IDIs yielded rich data, the group dynamic which can foster generation of innovation ideas is lost with this approach. In addition, we recruited participants from a single academic medical center. Barriers women face utilizing 17-P and suggested interventions may differ among women in other geographic areas.

Despite these limitations, our findings represent one of the first studies of potential interventions to overcome barriers to 17-P utilization that incorporates patient perceptions. Based on our analysis of the data it was clear that many participants were unaware of current standard practices such as appointment reminders and home injections. This observation suggests a need for interventions that ensure the current clinic practices are clearly communicated to patients. Based on the desire for injections outside the clinic, home-health aids or other health care extenders trained to provide subcutaneous or intramuscular injections may be efficacious for 17-P delivery.

## Conclusions

In conclusion, studies estimate that optimal 17-P utilization would decrease the number of PTBs by 10,000 infants annually translating to a significant decrease in infant morbidity and mortality and health care costs.^15–17^ Given the alarming and increasing rates of prematurity and PTB disparities, it is imperative as next steps to test, refine, and incorporate effective interventions into clinical practice.

## Availability of Data and Materials

The datasets generated and/or analyzed during the current study are not publicly available given ongoing analysis and documentation of findings related to additional research questions. Data are available from the corresponding author on reasonable request.

## Abbreviations Used

17-P: 17-hydroxyprogestrone caproate
FGD: focus group discussion
IDI: in-depth interview
NHB: non-Hispanic black
PTB: preterm birth
